# Oxytocin ameliorates lipopolysaccharide-induced acute orchitis model: interplay of oxidative stress and inflammatory pathways

**DOI:** 10.3389/fphar.2024.1506777

**Published:** 2025-01-07

**Authors:** Mohamed El-Sherbiny, Hany A. Elkattawy, Shimaa Hadhoud, Ahmed N. Nasr, Ateya M. Ibrahim, Omar Z. Ameer, Noorhan Alsaleebi, Joud Asfari, Madaniah O. Zakari, Moaz A. Mojaddidi, Ehab K. Ali, Hailah M. Almohaimeed, Ahmed Abdeen, Sahar K. Ali, Mamdouh Eldesoqui

**Affiliations:** ^1^ Department of Basic Medical Sciences, College of Medicine, AlMaarefa University, Riyadh, Saudi Arabia; ^2^ Department of Physiology, Faculty of Medicine, Zagazig University, Zagazig, Egypt; ^3^ Department of Anatomy, Faculty of Medicine, Mansoura University, Mansoura, Egypt; ^4^ College of Nursing, Prince Sattam bin Abdulaziz University, Al-Kharj, Saudi Arabia; ^5^ Department of Family and Community Health Nursing, Faculty of Nursing, Port Said University, Port Said, Egypt; ^6^ Department of Pharmaceutical Sciences, College of Pharmacy, Alfaisal University, Riyadh, Saudi Arabia; ^7^ Department of Basic Medical Sciences, College of Medicine, Taibah University, Medinah, Saudi Arabia; ^8^ Department of Anatomy and Embryology, Faculty of Medicine, AL-Azhar University, New Damietta, Egypt; ^9^ Department of Basic Sciences, College of Medicine, Princess Nourah bint Abdulrahman University, Riyadh, Saudi Arabia; ^10^ Department of Forensic Medicine and Toxicology, Faculty of Veterinary Medicine, Benha University, Toukh, Egypt; ^11^ Department of Medical Pharmacology, Faculty of Medicine, Zagazig University, Zagazig, Egypt

**Keywords:** testicular injury, hormonal therapy, oxidative stress, antioxidant, inflammation, sperm

## Abstract

**Introduction:**

Lipopolysaccharide (LPS), a constituent of the outer membrane of Gram-negative bacteria, is a powerful inducer of systemic inflammation and has been extensively utilized in experimental models to simulate inflammatory responses and septic disorders. Recent research indicates that oxytocin (OXY), a neuropeptide typically linked to social bonding and reproductive functions, may influence inflammatory processes. This work examines the impact of OXY on LPS-induced testicular damage, aiming to elucidate its therapeutic potential in addressing inflammatory disorders and broadening the comprehension of its functions beyond conventional neuroendocrine roles.

**Methods:**

Eighteen male albino rats were divided into three groups; the control group received no treatment; the LPS group received 0.5 mL of saline solution containing 5 mg/kg LPS intraperitoneally (orchitis model); and the LPS + OXY group received LPS and OXY (0.1 mg/kg) intraperitoneally every 12 h for 72 h.

**Results and discussion:**

Animals subjected to LPS were found to have severe orchitis, as evidenced by increased oxidative stress and surging inflammatory mediators (TNF-α, IL-1β, and IL-6), with declined IL-10 levels. Besides, LPS increased the malondialdehyde (MDA) and decreased the glutathione (GSH) levels, inducing an oxidative stress cascade. In addition, there are dramatic increases in the TLR4, MyD88, NF-κB, and PK2/PKR1 protein expression levels. All these events could alter the sperm count, morphology, and testicular architecture.

**Conclusion:**

Interestingly, OXY could mitigate LPS-induced oxidative damage and inflammation in testicular tissue alongside restoring the disrupted sperm count, motility, and morphology. This therapeutic potential of OXY might be accounted for by its anti-inflammatory, antioxidant, and antiapoptotic activities.

## 1 Introduction

Up to 10%–15% of couples worldwide struggle with infertility, and 50% of those cases are linked to a male component (Leslie et al., 2024). Men become infertile for three main reasons: hypogonadism caused by hypothalamic-pituitary abnormity, seminal outflow impairment, and dysfunctional testicles (Shen et al., 2022). Orchitis is a common inflammatory reproductive disorder that results in male infertility and a deterioration in sperm quality (Guo et al., 2024), and impairs spermatogenesis resulting in significant harm to both human and animal reproduction, as well as substantial economic losses in animal breeding (Theas et al., 2018). Numerous factors contribute to orchitis, including bacterial and viral infections as well as other conditions such as autoimmune diseases (Klein et al., 2016) and obesity (Leisegang et al., 2019). A male reproductive system infection or inflammation accounts for 13%–15% of occurrences of male infertility (Balló et al., 2023). Orchitis is characterized by intercellular lymphocytic infiltrations, damaging the seminiferous tubules. In extreme cases, this may completely arrest the process of sperm development (Fijak et al., 2011).

It is known that the pathophysiology of orchitis involves oxidative stress, apoptosis, and an imbalance in inflammatory cytokines (Petrella et al., 2022). Orchitis may be induced by discrepancies in the inflammatory and anti-inflammatory cytokine levels in testicular cells, including TNF-α, interleukin (IL)-1β, (IL-6), and IL-10 (Inoue et al., 2020; Chen et al., 2017). In the same context, the production of reactive oxygen species (ROS) by macrophages is a crucial factor contributing to inflammation and cell death in testicular tissue (Inoue et al., 2020). Thus, eliminating testicular oxidative stress and apoptotic damage and balancing inflammatory cytokines has gained global attention for the management of orchitis (Rival et al., 2006). Lipopolysaccharides (LPS) is a key element of the external membrane found in Gram-negative bacteria and can cause orchitis by promoting the production of ROS and inflammatory mediators such as TNF-α, IL-6, and IL-1β in testicular tissue (Zhu et al., 2022). Consequently, these events result in diminished sperm production, interruption of hormone synthesis in the testes, and compromise of the blood testis barrier in Sertoli cells (Shen et al., 2022; Zhu et al., 2022).

OXY is a hormone produced in the supraoptic and paraventricular hypothalamic nuclei and is referred to as a neuro-pituitary hormone (Matsuura et al., 2015). It facilitates the expulsion of milk during breastfeeding and the contraction of the uterus during childbirth (Uvnäs-Moberg et al., 2019). It has a fundamental role in adjusting a broad spectrum of physiological activities, such as appetite, pain perception, emotion, and social behavior (Jurek and Neumann, 2018). OXY enhances testosterone synthesis via Leydig cells and promotes spermiation (Frayne et al., 1996; Thackare et al., 2006). Ghasemnezhad et al. (2015) established a testicular damage model induced by ischemia-reperfusion in rats; thereby, OXY has shown a therapeutic potential where it could improve spermatogenesis, raise the Johnson score, and lower the apoptotic index.

OXY has been shown to exhibit antioxidative, antiapoptotic, and anti-inflammatory characteristics in sepsis-associated organ damage (Mehdi et al., 2022). OXY could suppress the synthesis of pro-inflammatory mediators in microglia triggered by lipopolysaccharides (LPS) in mouse brain via interaction with mitogen-activated protein kinases (MAPKs) pathway and inhibition of the LPS-stimulated secretion of TNF-α and IL-1β in these microglial cells (Yuan et al., 2016). OXY diminished macrophage sensitivity to LPS stimulation, resulting in reduced expression of inflammatory cytokines such as IL-1β, IL-6, and TNF-α, while simultaneously augmenting sensitivity to IL-4 stimulation, leading to enhanced expression of M2-type genes accompanied by suppression of NF-κB signaling (Tang et al., 2019).

Since orchitis significantly contributes to male animal reproductive problems, developing medicines that may prevent and cure it promptly is imperative. The present research aimed to investigate the potential therapeutic benefits of OXY against LPS-induced orchitis in a rat model.

## 2 Materials and methods

### 2.1 Ethical approval

The Ethics Committee for Animal Experimentation guidelines at Zagazig University’s College of Medicine approved the research protocol and the animal studies (Approval number: ZU-IACUC/3/F/244/2022). The NIH Guide for the Care and Use of Laboratory Animals was followed throughout the handling and scarification of the animals.

### 2.2 Animals

Eighteen adult male albino rats weighing 180 and 200 g and aged 7–8 weeks were purchased from Zagazig University, Egypt. All animals were kept in stainless-steel mesh-bottomed cages in the Department of Physiology, Faculty of Medicine, Zagazig University, in aseptic conditions, with a temperature range from 20°C to 24°C, suitable humidity (50%–60%), and a 12:12 h light/dark cycle. Throughout the trial, rats had unrestricted access to water and a balanced ration.

### 2.3 Research design

Following acclimatization, lasting 2 weeks, the rats were split randomly into three groups, six rats in each group. Group I (Control group) rats had no treatment; group II (LPS group), this group was only assigned to establish the acute orchitis model by injecting 0.5 mL saline containing LPS (Sigma-Aldrich, MO, United States) at a dosage of 5 mg/kg intraperitoneally (Chen et al., 2016), followed by a dose of physiological saline (20 mL/kg, intraperitoneally every 12 h); and group III (LPS + OXY group), rats received LPS followed by OXY (ApexBio, TX, United States) administrated at a dosage of 0.1 mg/kg by intraperitoneal injection every 12 h for 72 h (Sever et al., 2021).

After 24 h of the last treatment, the rats were weighed, and blood samples were collected from the retroorbital venous plexus under anesthesia (ketamine, 50 mg/kg, and xylazine, 2.20 mg/kg) (Ghasemnezhad et al., 2015). Following centrifugation of blood for 15 min at 3,000 rpm, serum was separated and preserved at −20°C for further biochemistry tests. Next, animals were euthanized after an overdose of anesthesia, and orchidectomy was performed using the open castration approach; briefly, the groin and scrotum were shaved aseptically and then under sterile conditions, a low midline incision was performed, and the spermatic cords were bilaterally identified to expose the testicles. The spermatic cord is subsequently clamped and ligated, followed by the excision of the testicle. The two testes were then dissected and weighed. The cauda epididymis was also collected for analysis of semen. The percentage ratio of testicular weight to total body weight was obtained to calculate the relative testicular weight. One testis was stored at −80°C for subsequent detection of oxidative stress, inflammatory mediators, and Western blotting, while the other was used for histopathological evaluation.

### 2.4 Semen examination

Semen collection was performed by longitudinally opening the cauda epididymis, and sperm motility was examined microscopically. The sperm cells that showed some movements were classified as motile, whereas those that showed no movement were classified as non-motile (Han et al., 2019). Furthermore, a hemocytometer with the improved Neubauer chamber (LABART^®^, New Delhi, India) was used for sperm counting (Ebokaiwe et al., 2018). The spermatic suspensions were stained with 1% eosin Y solution at a 10:1 ratio and analyzed under a light microscope to evaluate sperm morphology. Each rat provided 200 spermatozoa, which were analyzed for morphological alterations. The sperm percentage with normal morphology was noted (Ekaluo et al., 2009).

### 2.5 Evaluation of serum testosterone level

The serum level of testosterone hormone was evaluated by an ELISA kit provided by BioCheck, Inc., Foster City, United States, in compliance with the manufacturer’s guidelines.

### 2.6 Inflammatory and oxidative stress markers assessment in testicular tissue

Testicular specimens were standardized in a pH 7.4 solution containing 50 mM potassium phosphate and then centrifuged for 15 min. The resulting homogenate was then employed to evaluate the MDA and GSH concentrations using commercial colorimetric kits (Laboratory Biodiagnostics, Cairo, Egypt). Additionally, rat ELISA kits (Laboratory Biodiagnostics) were used to determine the inflammatory markers (TNF-α, IL-1β, IL-6, and IL-10) in compliance with the manufacturer’s guidelines.

### 2.7 Histopathological analysis

The testicular specimens were submerged in Bouin’s solution, then paraffinized, sectioned into five μm-thick slices, and finally stained with hematoxylin and eosin (H&E). The sections were scrutinized using a light microscope, and their histological picture was evaluated. Spermatogenesis was categorized according to Johnsen’s score (Johnsen, 1970). This scoring system assigns a score from 1 to 10 to each tubular cross section based on specific criteria: score = 10 indicated full spermatogenesis and optimal tubules; score = 9 indicated the presence of many spermatozoa with disrupted spermatogenesis; score = 8 indicated a low number of spermatozoa, while a score = 7 suggested a lack of spermatozoa but the presence of many spermatids. A score of 6 signifies a low number of spermatids; a score = 5 denotes the absence of spermatozoa or spermatids accompanied by a substantial presence of spermatocytes; a score = 4 reflects a low number of spermatocytes, while a score = 3 shows the existence of just spermatogonia. A score of 2 implies the absence of germ cells but the preservation of Sertoli cells. Lastly, a score of 1 signifies the lack of germ and Sertoli cells.

### 2.8 Immunohistochemical approach

Five µm-thick sections were obtained using paraffin blocks. After that, these sections were rehydrated, deparaffinized, and incubated for 15 min in a 3% H_2_O_2_/methanol solution to stop the endogenous peroxidase activity. Afterward, the slides were subjected to heat treatment in citrate buffer at a temperature of 95°C for 10 min to enhance the process of antigen retrieval, after which they were allowed to cool for an hour and then washed three times; sections were cleaned for 5 min each using phosphate-buffered saline (PBS). The primary antibodies of MyD88 (Rabbit pAb, ABclonal, Wuhan, China, Cat# A0786, dilution 1:100), NF-κB (Rabbit pAb, ABclonal, Cat# A3108, dilution 1:100) and TLR4 (Mouse mAb, Servicebio, Wuhan, China, Cat# GB12186, dilution 1:1,000) were added to the tissue section and incubated overnight at four°C. The slides were then treated with a rabbit-mouse secondary antibody (BIO SB Inc., CA, United States, Cat# BSB 0268) for 45 min. A 3,3-diaminobenzidine tetrahydrochloride (DAB) kit was used to detect immunoreactivity colorimetrically (Eldesoqui et al., 2023). The sections were then counterstained with hematoxylin. Positive staining was indicated by dark brown areas in the cytoplasm or nucleus under a light microscope supported with a digital camera system.

### 2.9 Morphometry and image analysis

Five randomly chosen portions from each animal in every group were examined. For valid comparisons, the uniformity of section thickness was considered. An Olympus model BX53 light microscope (Olympus, Tokyo, Japan) linked to an Olympus^®^ SC100 digital camera (Olympus Soft Imaging Solutions GmbH, Munster, Germany) was utilized to collect the photos. In the H&E-stained sections, Various parameters, such as surface area and diameter of the seminiferous tubules (STs), as well as the height of their epithelium, were evaluated by ImageJ software (National Institute of Health, Bethesda, MD, United States) (Sayed et al., 2023). In addition, the immunopositive stained area percentages for MYD88, TLR4, and NF-κB were evaluated using the same software applying the color deconvolution plugin and H-DAB vector (Eldesoqui et al., 2022).

### 2.10 Immunoblotting

A tissue homogenate was prepared from the testicular tissue, and the protein content was ascertained using the Bradford Protein Assay kit (Bio Basics Inc., Ontario, Canada, Cat# SK3041). The proteins were subsequently segregated on SDS-PAGE (sodium dodecyl sulfate-polyacrylamide gel electrophoresis) using a stacking and a resolving gel. After separation, all proteins were blotted in a BioRad Trans-Blot Turbo system onto a polyvinylidene difluoride membrane. Next, tris-buffered saline with Tween 20 (TBST) and 3% bovine serum albumin was applied for membrane block at room temperature for 1 h. The membranes were then treated overnight at 4°C with the primary antibodies for rabbit polyclonal anti-PK2 (Abcam, MA, United States, Cat# ab87360, dilution 1: 1,000), rabbit polyclonal anti-PKR1 (BT LAB, Shanghai, China, Cat# BT-AP12193, dilution 1: 1,000), and mouse monoclonal anti-B-actin (Abcam, Cat# ab227387, dilution 1:2000). After washing the blot, it was incubated with a secondary antibody (goat anti-rabbit IgG-HRP-1mg Goat mab; Novus Biologicals, CO, United States, Cat# HAF008) for an hour at room temperature. Protein bands were visualized by exposure to a chemiluminescent substrate (Clarity™ Western ECL substrate, Bio-Rad, CA, United States, Cat# 170–5060); thereby, the BioRAD ChemiDoc Imaging System (Bio-Rad) was employed to detect the bands. Then, NIH ImageJ (National Institutes of Health (NIH), MD, United States) software was used to quantify the band intensities.

### 2.11 Statistical analyses

Shapiro-Wilk’s test was used to assess the normality of the variables used and whether they were normally distributed at *P* > 0.05. Multiple comparisons were assessed using one-way analyses of variance (ANOVA), followed by Tukey’s *post hoc*. Kruskal–Wallis, followed by Dunn’s test, was applied for statistical analysis of Johnsen’s score between groups. Data statistics and visualization were conducted by OriginPro 2019b (OriginLab Corporation, MA, United States). RStudio software; version 2023.12.0 “Ocean Storm” Release (33206f75, 2023-12-17) for Windows under R version 4.0.2. was employed to perform the multivariate analyses.

## 3 Results

### 3.1 Impact of LPS and/or OXY on relative testicular weight, testosterone level, oxidative stress markers

**FIGURE 1:**
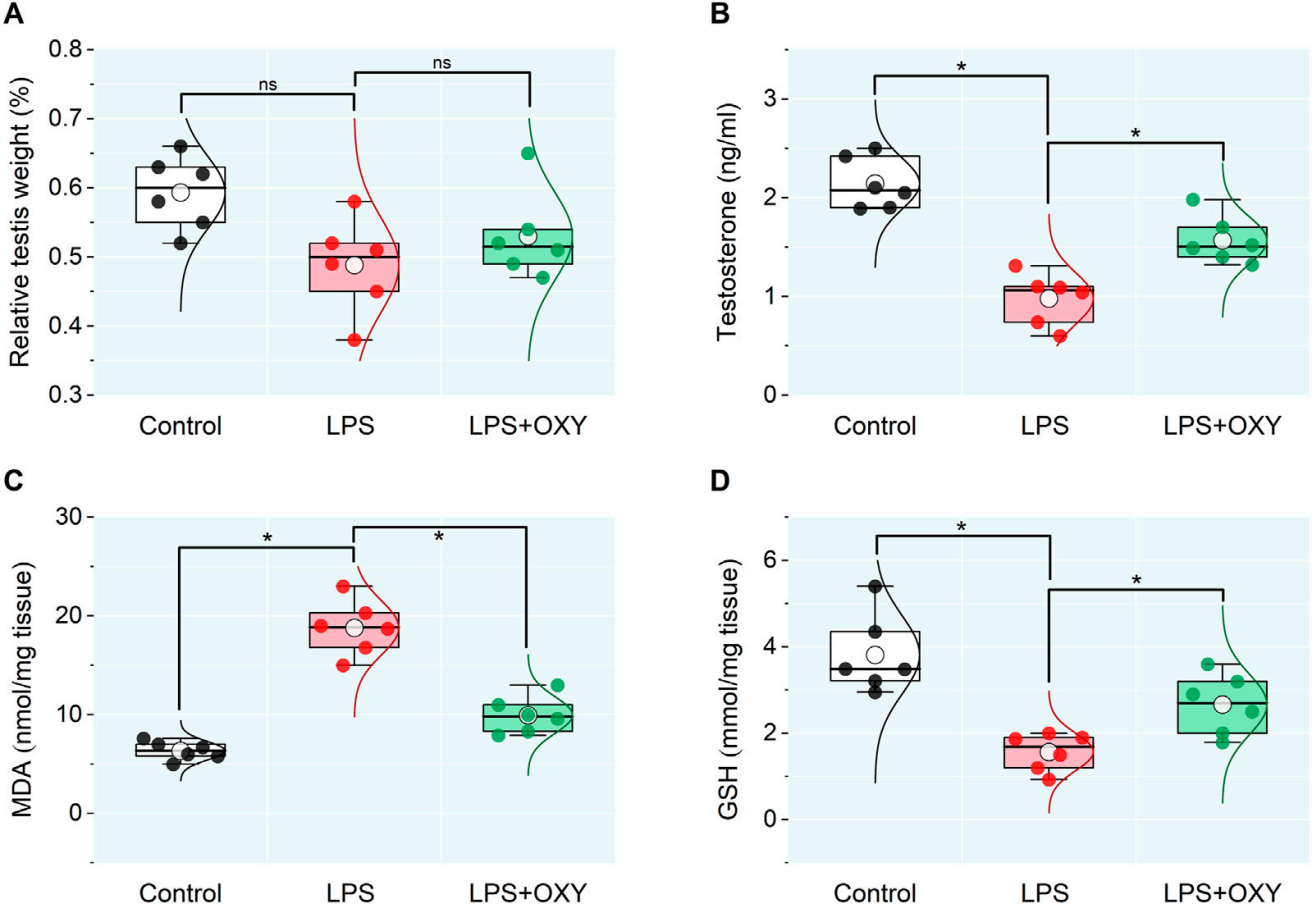
Dot-box plot presents the effect of oxytocin on the testicular weight relative to body weight **(A)**, serum testosterone **(B)**, and oxidative stress markers **(C, D)**. LPS, lipopolysaccharides; GSH, glutathione; MDA, malondialdehyde; OXY, oxytocin. Data are presented as mean ± SE (n = 6); *P < 0.05.

The data presented in Figure 1 indicated a reduction in the relative testicular weight in the LPS-exposed group compared to controls which was nonsignificant, however, testosterone level in the LPS group recorded a remarkable decrease relative to the control rats. This reduction in the testosterone level was alleviated after the OXY administration. Furthermore, compared to controls, the LPS group rats showed significantly higher testicular MDA and lower GSH levels; thereby, adminstartion of OXY could greatly attenuate these actions.

### 3.2 Effect of LPS and/or OXY on the testicular inflammatory cytokines

**FIGURE 2:**
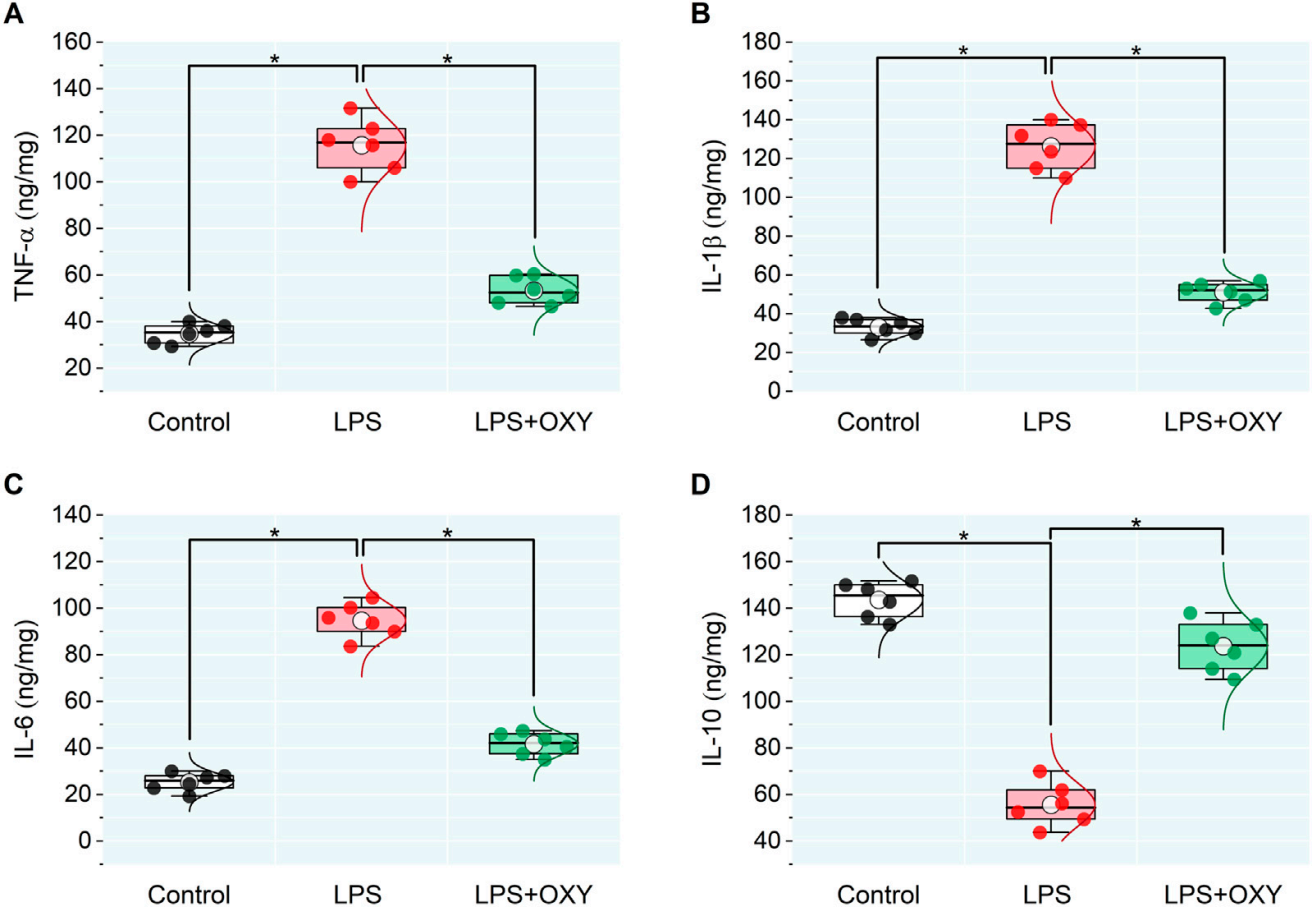
Dot-box plot presents the effect of oxytocin on inflammatory cytokines in testicular tissue, including TNF-α **(A)**, IL-1β **(B)**, IL-6 **(C)**, and IL-10 **(D)**. Data are presented as mean ± SE (n = 6); *P < 0.05 IL, interleukin; LPS, lipopolysaccharides; OXY, oxytocin; TNF-α, tumor necrosis factor Alpha.

In the LPS-injured group, there was a large upsurge in the testicular pro-inflammatory mediators TNF-α, IL-1β, and IL-6 along with a significant drop in IL-10. When OXY was administered, relative to the LPS group, there was a noticeable decrease in the pro-inflammatory cytokines and enhancement in the anti-inflammatory cytokine, IL-10 (Figure 2).

### 3.3 Effect of LPS and/or OXY on semen quality

**FIGURE 3:**
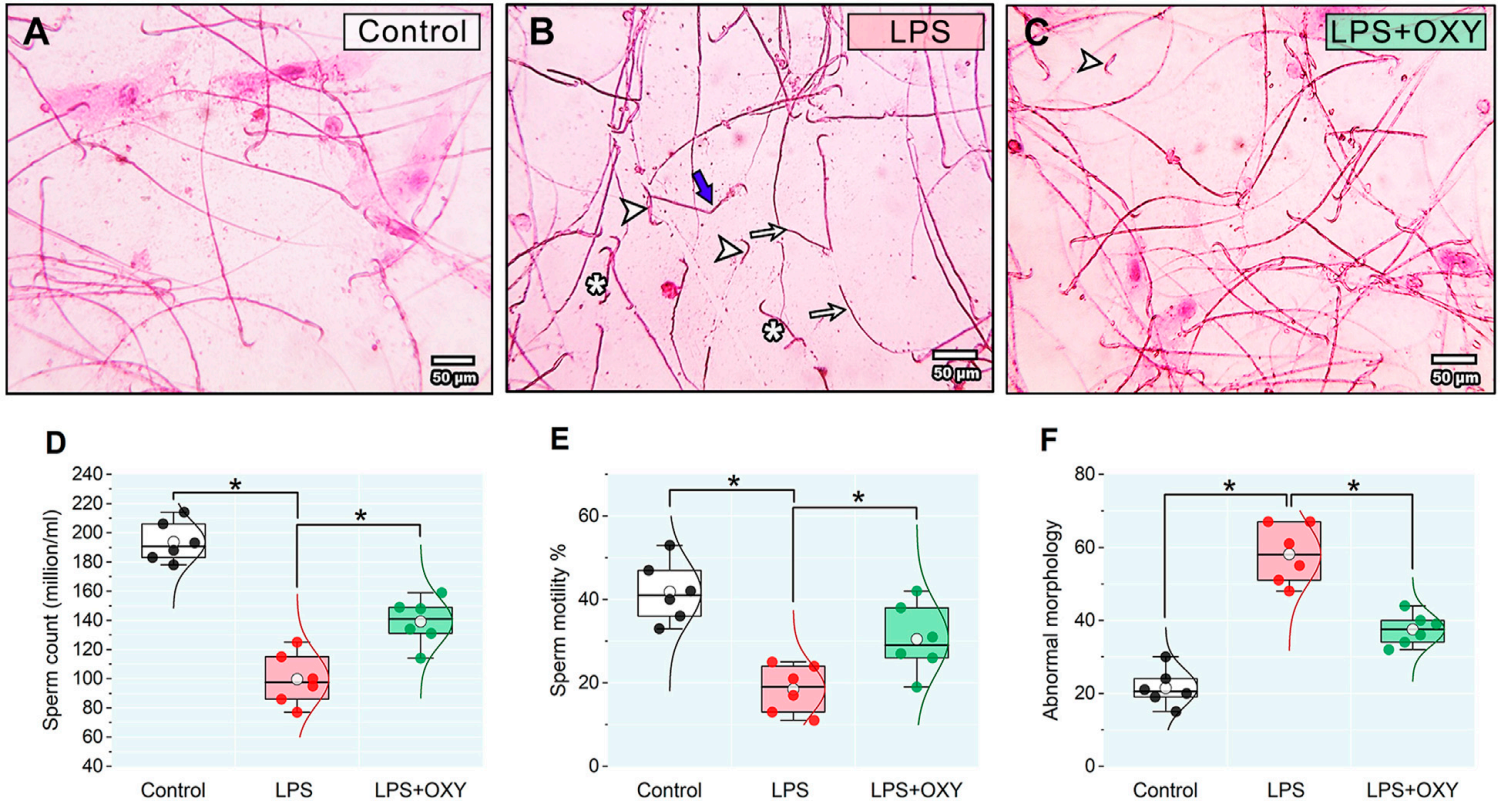
Effect of LPS and/or OXY on semen qualities. Semen smear stained with 0.05% aqueous solution of eosin-Y illustrating morphologically normal sperm under the light microscope in the control normal group **(A)**. Various severe sperm defects are observed in semen smears from the LPS group, including detached heads (arrowhead), many dwarf sperms (*), headless sperms (arrows), and bent necks (thick blue arrows) **(B)**. Semen smears from the LPS + OXY group showed mild sperm defects, including a few detached heads (arrowhead) **(C)**. Dot-box plots present sperm count **(D)**, sperm motility % **(E)**, and Abnormal morphology **(F)**. Bars = 50 µm. LPS, lipopolysaccharides; OXY, oxytocin. Data are presented as mean ± SE (n = 6); *P < 0.05.

As illustrated in Figure 3, LPS elicited considerable decreases in both sperm count and motility; also, a discernible rise in the proportion of aberrant morphology was noted relative to the control group. On the other hand, when compared to rats in the LPS group, OXY treatment increased sperm count and motility and decreased sperm abnormalities.

### 3.4 Effect of LPS and/or OXY on the histological architecture

**FIGURE 4:**
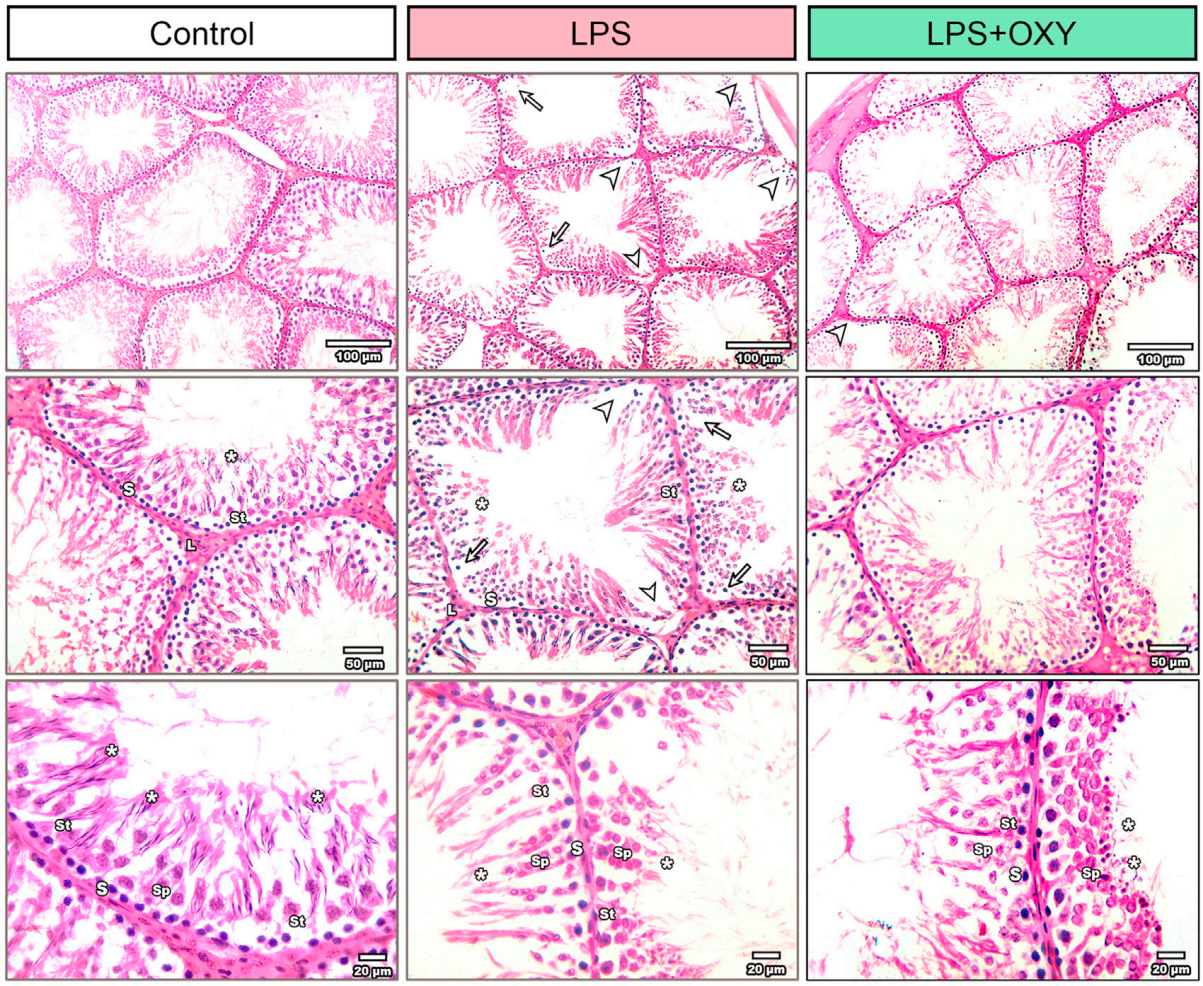
Effect of LPS and/or OXY on testicular architecture. Testicular sections from the control group show normal crossly sectioned seminiferous tubules with minimal interstitial tissue containing cells of Leydig. Higher magnification (Bar = 20 µm) shows lining epithelium consisting of spermatogonia (s), spermatocytes (sp), and spermatids (*) attached to Sertoli cells (st). Testicular sections from the LPS group showed multifocal loss of tubular epithelium (arrowhead) with vacuolation (arrow) and decreased numbers of spermatids (*). Testicular sections from the LPS + OXY group show improved histology of tubular epithelium with still few numbers of spermatids (*). Each group was present at three magnification levels (Bars = 100, 50, 20 µm). LPS, lipopolysaccharides; OXY, oxytocin.

As seen in Figure 4, the testis from the control rats showed normal morphology of seminiferous tubules surrounded by well-defined basal lamina. It exhibited normal stages of spermatogenesis separated by interstitial tissue. The LPS-injured testis exhibited a distorted architecture of seminiferous tubules in the form of focal loss of tubular epithelium and vacuolated cells with a decreased number of spermatids. Additionally, as shown in Table 1, the LPS group displayed a substantial decrease in the seminiferous epithelium height relative to the control group, with no significant reduction of seminiferous tubule diameter and surface area. OXY improved the histology of tubular epithelium with few numbers of spermatids. Likewise, compared to the LPS group, OXY raised the height of seminiferous epithelium, but it was still significantly lower than that of the controls. The Jhonson’s score of the LPS-intoxicated rats showed a significant decline upon the administration of OXY (Figure 4; Table 1).

**TABLE 1:** Effect of LPS and/or OXY on Johnson’s score and morphometry of testis.

Item	Control	LPS	LPS + OXY
Seminiferous tubules diameter (µm)	191.3 ± 5.92	187.8 ± 10.87	181.1 ± 8.12
Seminiferous tubules surface area (µm^2^)	44,339 ± 3,320	38,231 ± 2,583	38,383 ± 2,458
Seminiferous epithelium height (µm)	112.20 ± 6.77	61.20 ± 4.15*	81.50 ± 4.68*^#^
Johnsen’s score	9.62 ± 0.18	6.87 ± 0.12*	8.375 ± 0.18

Data are presented as mean ± SE.

*P < 0.05 vs. Control group; ^#^ *P* < 0.05 vs. LPS, group.

### 3.5 MyD88, TLR4, and NF-κB proteins expression in testicular tissue

**FIGURE 5:**
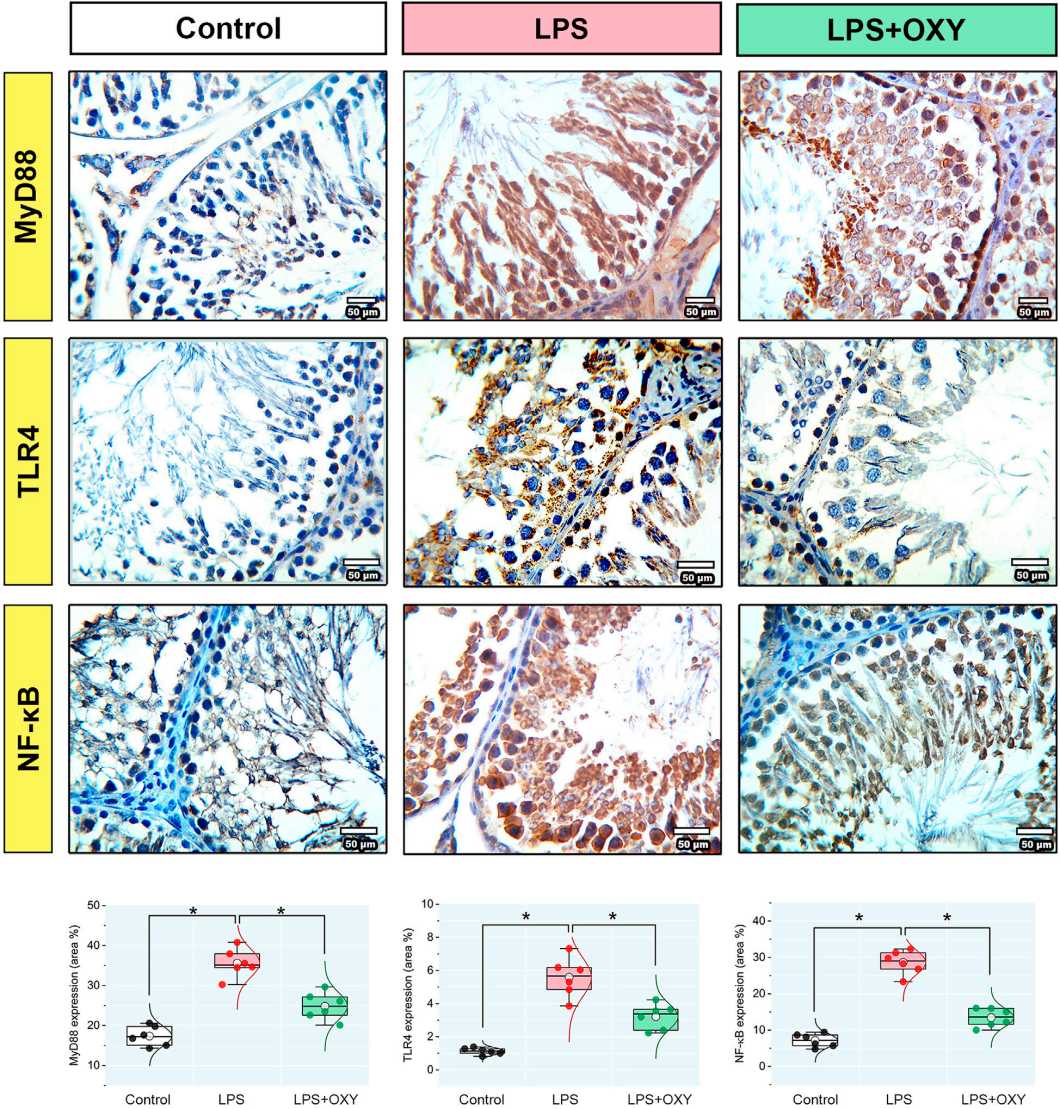
Effect of LPS and/or OXY on MyD88, TLR4, and NF-κB protein expressions in testicular tissue. Dot-box plots present the positive area %. LPS, lipopolysaccharides; MyD88, myeloid differentiation primary response 88; NF-κB, Nuclear factor-kappa B; OXY, oxytocin; TLR4, toll-like receptor 4. Data are presented as mean ± SE (n = 6); *P < 0.05.

Dramatic upregulations in the expression levels of MyD88, TLR4, and NF-κB (Figure 5) were observed in the LPS-insulted rats about the control rats; these elevations were diminished when OXY was co-administrated with LPS.

### 3.6 Expression levels of PK2 and PKR1 proteins in testicular tissue

**FIGURE 6:**
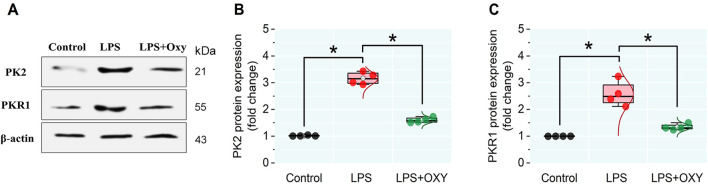
Effect of LPS and/or OXY on PK2 and PKR1 protein expressions in testicular tissue. **(A)** immunoblots of testicular PK2 and PKR1. **(B, C)** Semi-quantification of PK2 and PKR1 proteins, respectively. LPS, lipopolysaccharides; OXY, oxytocin; PK2, Prokineticin 2; PKR1, Prokineticin receptor 1. Data are presented as mean ± SE (n = 4); *P < 0.05.

LPS provoked remarkable increases in the PK2 and PKR1 protein expressions in testicular tissue compared to control animals. However, these elevations were significantly reduced in the LPS + OXY group (Figure 6).

### 3.7 Multivariate analyses

**FIGURE 7:**
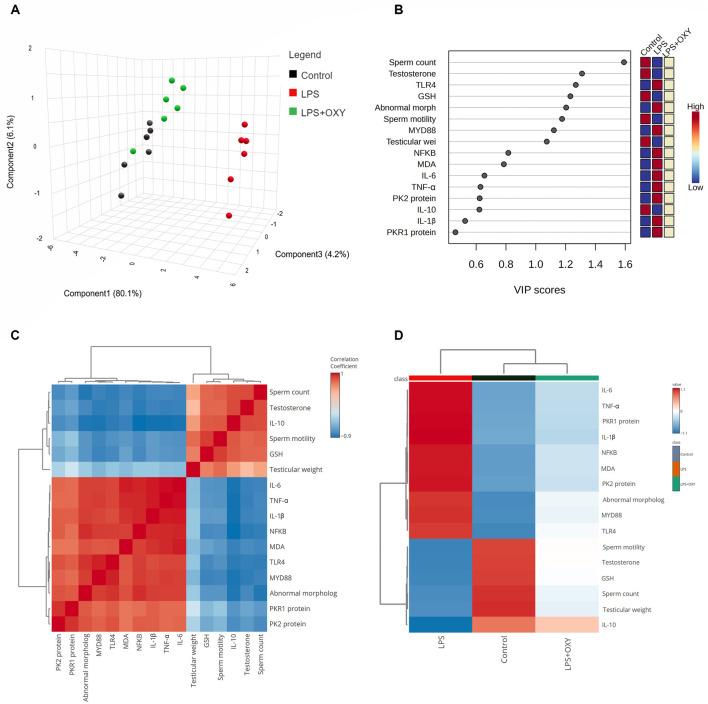
Multivariate analyses of all data sets after LPS and/or OXY intervention. **(A)** 3D plot of PCA for discriminating all interventions. **(B)** VIP scores declare the most influencing variable. **(C)** Correlation heatmap illustrates the inter-relationship among studied variables. **(D)** Clustering heatmap indicates the concentration values of tested parameters among different groups. GSH, glutathione; IL, interleukin; LPS, lipopolysaccharides; MDA, malondialdehyde; MyD88, myeloid differentiation primary response 88; NF-κB, Nuclear factor-kappa B; OXY, oxytocin; PCA, principal component analysis; PK2, Prokineticin 2; PKR1, Prokineticin receptor 1; TLR4, toll-like receptor 4; TNF-α, tumor necrosis factor Alpha; VIP, Variable importance in projection.

As depicted in Figure 7A, principal component analysis (PCA) was applied to determine the relationship among the different interventions. The PCA revealed three principal dimensional elements for all measured parameters with a total of 90.4% of the variation. Component 1 exhibited the most dominant proportion of variation (80.1%). However, components 2 and 3 accounted for a lower proportion of 6.2% and 4.2%, respectively, compared to component 1. The data obtained by PCA revealed that the LPS-intoxicated rats were distinctively separated from the control and LPS + OXY groups on the opposite side. Based on these results, animals that received both LPS and OXY displayed a remarkable difference compared to those injured solely with LPS. As seen in Figure 7B, the variable importance in projection (VIP) scores revealed that sperm qualities (sperm count, morphology, and motility), oxidative stress (MDA), and inflammatory markers (IL-6, TNF-α, IL-1β, and NF-κβ) were the most influencing variables in the present study. The correlation heatmap between all variables provided that either measured parameters were positively or negatively correlated (Figure 7C). Besides, the clustering heatmap shown in Figure 7D provided an intuitive visual representation of all the data. It highlighted the key differences in concentration levels of all variables among different treated groups. According to the heatmap, rats exposed to LPS were likelier to show greater testicular damage. Alternatively, rats responded well to the protective effect of OXY therapy when it was co-administered with LPS.

## 4 Discussion

Infections and inflammation can negatively impact male fertility in the body and testes, which can result in a decrease in sperm production that may not recover even with antibiotic therapy (Li et al., 2023). Inflammatory cytokine imbalance influences spermatogenesis by altering cell-to-cell interactions, producing reactive oxygen species (ROS), and activating Toll-like receptors, potentially leading to male infertility (Syriou et al., 2018). In response to LPS insult, severe inflammatory reactions are induced, liberating pro-inflammatory cytokines from testicular cells, including Sertoli and Leydig cells, leading to testicular dysfunction (Reddy et al., 2006; Takeda and Akira, 2015).

In the current study, serum testosterone levels and anti-inflammatory IL-10 were considerably lowered by LPS injection. Conversely, LPS injection elevated the TNF-α, IL-1β, and IL-6 levels. LPS also increased aberrant sperm morphology, decreased sperm motility, and decreased sperm count. The disproportion of pro- and anti-inflammatory mediators, including TNF-α, IL1β, IL-6, and IL-10, in testicular cells promotes the induction of acute orchitis (Chen et al., 2017; O’Bryan et al., 2000). Intraperitoneal LPS injection prompts acute inflammatory responses, inhibiting testicular steroidogenesis and producing male germ cell apoptosis (Reddy et al., 2006; Metukuri et al., 2010; Wang et al., 2019a). LPS-induced inflammation alters testicular physiology (Hedger and Meinhardt, 2003). Moreover, it stimulates the synthesis of TNF-α, IL-1β, and IL-6 (Reddy et al., 2006; Metukuri et al., 2010; Wang et al., 2019a). Elevated TNF-α levels might impede steroidogenesis under inflammatory conditions and induce germ-cell apoptosis in experimental autoimmune orchitis (Hong et al., 2004; Li et al., 2013; Theas et al., 2008). Furthermore, IL-6 and IL-1β inhibit testosterone production *by* suppressing the expression of steroidogenic enzymes (Wang et al., 2019a; Abarikwu et al., 2024; Huang et al., 2020).

The current results demonstrated that LPS-induced oxidative stress, which increased MDA, reduced the antioxidant defense by decreasing GSH. Oxidative stress and ROS overproduction significantly influence testicular impairment, which primarily involves damage to the germ and Leydig cells, along with reduced sperm vitality and testosterone levels (Metukuri et al., 2010; Abd-Allah et al., 2009; Al-Olayan et al., 2014). Previous studies reported that LPS caused excessive ROS generation, which set off a chain reaction of oxidative damage manifested as a marked drop in GSH concentration and a sharp rise in MDA levels (Reddy et al., 2006; Li et al., 2013; Allen et al., 2004).

There have been suggestions that inflammatory reactions may be related to oxidative damage. An essential function of the TLR4-MyD88 pathway is to produce inflammatory mediators (Kawai and Akira, 2010). The current investigation showed that LPS increased TLR4, MyD88, and NF-κB expression, enhancing the production of pro-inflammatory cytokines (TNF-α, IL-1β, and IL-6) which was in paralel with the previous reports (Tsubaki et al., 2015; Wang et al., 2019b; Mulero et al., 2019; Shen et al., 2020). In the present investigation, the rats injected with LPS exhibited upregulated PK2 and PKR1 expression levels. Consistent with earlier findings, exposure to LPS has resulted in elevated expressions of pro-inflammatory mediators, PK2, and PKR1 (Chen et al., 2016; Huang et al., 2020; Martucci et al., 2006). Testicular inflammation has been associated with PK2, which is mostly expressed in the testes, via the PK2/PKR1 cascade, which is implicated in the pathophysiology of the LPS-induced orchitis model (Chen et al., 2016; LeCouter and Ferrara, 2003). PK2 induces macrophage recruitment and the release of pro-inflammatory mediators (Martucci et al., 2006). Furthermore, the present results showed that the rats in the LPS group exhibited decreased IL-10. Previous research has shown that overexpression of PK2 weakens the immune system’s ability to fight inflammation by drastically lowering the production of IL-10 (Martucci et al., 2006; Franchi et al., 2008; Cook et al., 2010). These findings point to the significance of the PK2/PKR1 signal cascade in controlling the inflammatory response during infection. Histopathological analysis demonstrated the adverse effects of LPS on the testis, which included vacuolation, a decrease in the number of spermatids, and a focal loss of the tubular germinal epithelium. Other studies corroborated these findings (Chen et al., 2016; Wang et al., 2019a; Shen et al., 2020).

The present work demonstrated that OXY could mitigate the testicular damage in the LPS-induced acute orchitis model. OXY demonstrated these actions by reducing the levels of pro-inflammatory cytokines (TNF-α, IL-1β, and IL-6) and simultaneously boosting the anti-inflammatory response by elevating the IL-10 level. Additionally, OXY could improve the antioxidant ability of the testicles by raising the concentration of GSH and diminishing the level of MDA. Furthermore, our study has demonstrated OXY’s antioxidant and anti-inflammatory characteristics through its capacity to restore histological architecture and improve semen qualities. Herein, the administration of OXY improved sperm count and motility and reduced sperm morphological abnormalities. Besides, OXY markedly decreased the expression of immune-related proteins such as MyD88, TLR4, NF-κB, PK2, and PKR1. It's interesting to note that Leydig cells produce OXY, which may have a localized effect on testosterone synthesis. Additionally, it controls how the epididymis ducts and seminiferous tubules contract (Thackare et al., 2006; Nicholson et al., 1994).

It has been shown that exogenous OXY treatment decreases tissue pathology in several animal models (Houshmand et al., 2009; Akdemir et al., 2014; An et al., 2019). OXY has reportedly been proven to prevent inflammation induced by LPS in microglial cells (Yuan et al., 2016), and effectively guard against experimental colitis through the AKT/Pi3K pathway (Dou et al., 2021). The OXY receptor gene has response components for interleukins and acute-phase reactants; meanwhile, this receptor is upregulated in the LPS-activated macrophages (Szeto et al., 2017). OXY can decrease the release of pro-inflammatory cytokines like IL-1β and IL-6 and enhance anti-inflammatory cytokines like IL-4 and IL-10 (An et al., 2019). Surprisingly, in another study, OXY could be utilized to counteract COVID-19-induced inflammation due to its anti-inflammatory properties (Buemann et al., 2021). It was also reported that OXY attenuated acute lung injury induced by LPS in mice by inhibiting the TLR4/NLRP3/NF-κB inflammatory signaling cascade along with upregulation of OXY receptors in the alveolar macrophages (An et al., 2019). In addition, OXY could counteract the sepsis-induced flow in the pro-inflammatory cytokine TNF-α and block the shift of macrophages from a neutral to a pro-inflammatory state (Tang et al., 2019). When OXY binds to its receptor, it inhibits the NF-κB signaling pathway (Szeto et al., 2017) and, therefore diminishes the production of TNF-α and IL-6 (Tang et al., 2019; Buemann et al., 2021). According to earlier research, the OXY-treated torsion group had higher Johnsen scores than the testicular torsion group (Ghasemnezhad et al., 2015; Fırat et al., 2018). Moreover, OXY has a significant testicular vasodilator effect on the testicular vascular tone and steroid levels in rams (El-Shalofy and Hedia, 2021).

**FIGURE 8:**
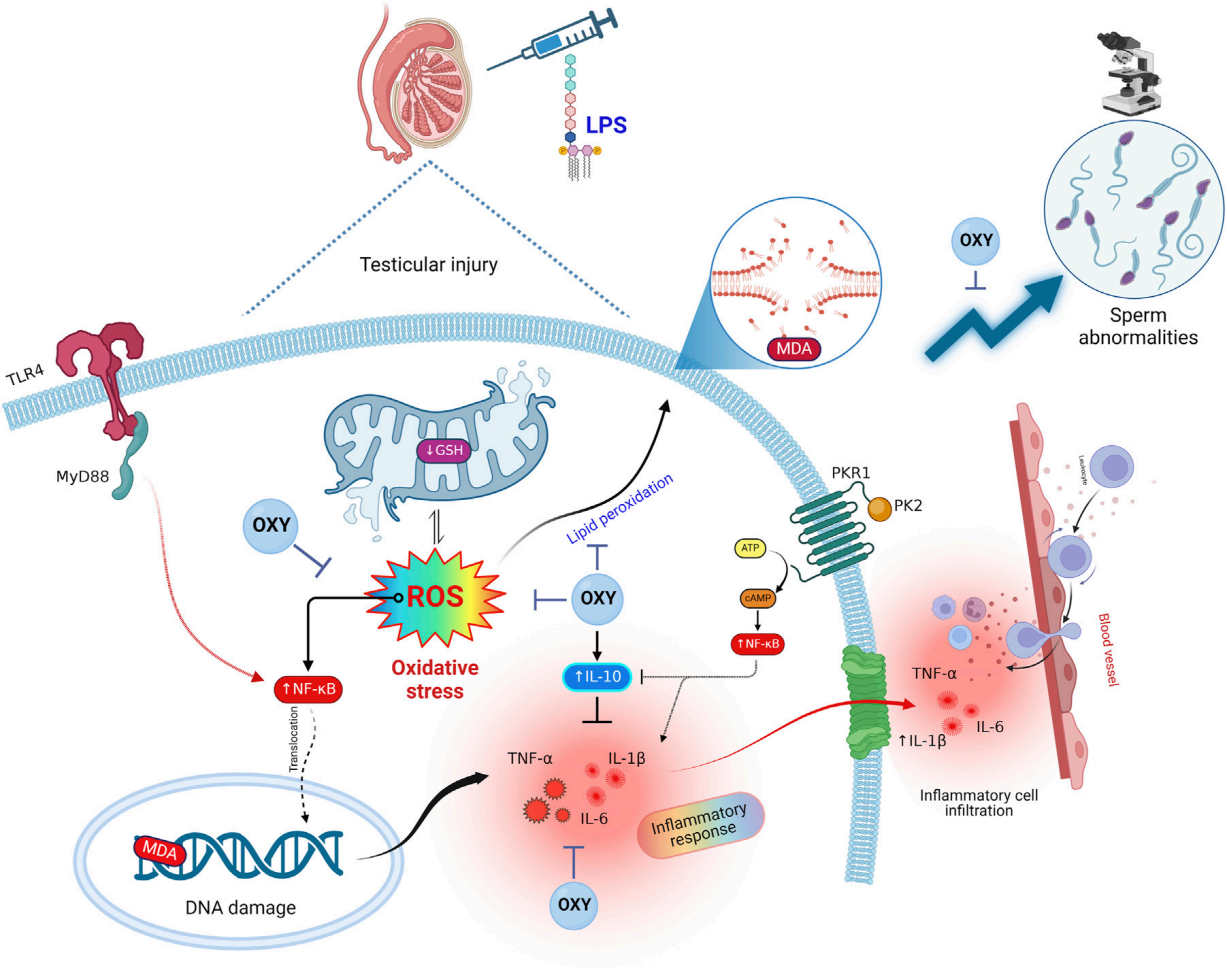
Proposed mechanisms located behind the pharmacological potential of OXY toward LPS-induced testicular injury. cAMP, cyclic adenosine monophosphate; GSH, glutathione; IL, interleukin; LPS, lipopolysaccharides; MDA, malondialdehyde; MyD88, myeloid differentiation primary response 88; NF-κB, Nuclear factor-kappa B; OXY, oxytocin; PCA, principal component analysis; PK2, Prokineticin 2; PKR1, Prokineticin receptor 1; ROS, reactive oxygen species; TLR4, toll-like receptor 4; TNF-α, tumor necrosis factor Alpha; VIP, Variable importance in projection.

Interestingly, in light of all the previously described findings, the PCA data demonstrate the extent of testicular damage caused by exposure to LPS, with the toxic group being able to be distinguished from the control or LPS + OXY groups. Similarly, since the LPS + OXY group is situated near the control group along component 1, the overall statistics show that OXY has a large preventative impact. In addition, the VIP scores affirmed that inflammation and oxidative stress were the main modulators in the LPS-induced testicular damage; thereby, the OXY could counteract such toxic actions of LPS. Meanwhile, the correlation heatmap attested to a significant correct correlation between all studied parameters, which supported all the above-obtained data. In the same data frame, the difference in each variable’s concentration levels across the various treatment groups was indicated by the clustering heatmap. According to the heatmap, rats exposed to LPS were more likely to show greater testicular damage. Alternatively, rats responded well to the protective effect of OXY therapy when it was co-administered with LPS. The proposed mechanisms behind the pharmacological potential of OXY toward LPS-induced testicular damage are summarized in Figure 8.

## 5 Conclusion

This study found that OXY effectively alleviates testicular dysfunction induced by LPS. This preferential action was achieved by mitigating the oxidative stress caused by LPS by enhancing GSH and reducing MDA tissue contents. OXY could also decrease pro-inflammatory cytokines levels and boost anti-inflammatory by downregulating TLR4, MyD88, NF-κB, and PK2/PKR1 pathways, which are linked to LPS-induced orchitis. In addition, OXY improves sperm count, motility, and morphology and maintains normal testicular histology. These results imply that OXY might be a viable curative option for testicular dysfunction and male infertility resulting from acute inflammatory disorders.

## Data Availability

The original contributions presented in the study are included in the article/supplementary material, further inquiries can be directed to the corresponding authors.

## References

[B1] Abarikwu S. O. Mgbudom-Okah C. J. Ndufeiya-Kumasi L. C. Monye V. E. Aruoren O. Ezim O. E. (2024). Influence of triazines and lipopolysaccharide coexposure on inflammatory response and histopathological changes in the testis and liver of BalB/c mice. Heliyon 10 (2), e24431. 10.1016/j.heliyon.2024.e24431 38293467 PMC10826326

[B2] Abd-Allah A. R. Helal G. K. Al-Yahya A. A. Aleisa A. M. Al-Rejaie S. S. Al-Bakheet S. A. (2009). Pro-inflammatory and oxidative stress pathways which compromise sperm motility and survival may be altered by L-carnitine. Oxidative Med. Cell. Longev. 2, 73–81. 10.4161/oxim.2.2.8177 PMC2763248 20357928

[B3] Akdemir A. Erbas O. Gode F. Ergenoglu M. Yeniel O. Oltulu F. (2014). Protective effect of oxytocin on ovarian ischemia-reperfusion injury in rats. Peptides 55, 126–130. 10.1016/j.peptides.2014.02.015 24630974

[B4] Allen J. A. Diemer T. Janus P. Hales K. H. Hales D. B. J. E. (2004). Bacterial endotoxin lipopolysaccharide and reactive oxygen species inhibit Leydig cell steroidogenesis via perturbation of mitochondria. 25:265–275. 10.1385/ENDO:25:3:265 15758255

[B5] Al-Olayan E. M. El-Khadragy M. F. Metwally D. M. Abdel Moneim A. E. (2014). Protective effects of pomegranate (Punica granatum) juice on testes against carbon tetrachloride intoxication in rats. BMC Complement. Altern. Med. 14, 164. 10.1186/1472-6882-14-164 24884677 PMC4041339

[B6] An X. Sun X. Hou Y. Yang X. Chen H. Zhang P. (2019). Protective effect of oxytocin on LPS-induced acute lung injury in mice. Sci. Rep. 9 (1), 2836. 10.1038/s41598-019-39349-1 30808956 PMC6391417

[B7] Balló A. Busznyákné Székvári K. Czétány P. Márk L. Török A. Szántó Á. (2023). Estrogenic and non-estrogenic disruptor effect of zearalenone on male reproduction: a review. Int. J. Mol. Sci. 24 (2), 1578. 10.3390/ijms24021578 36675103 PMC9862602

[B8] Buemann B. Marazziti D. Uvnäs-Moberg K. (2021). Can intravenous oxytocin infusion counteract hyperinflammation in COVID-19 infected patients? World J. Biol. Psychiatry 22 (5), 387–398. 10.1080/15622975.2020.1814408 32914674

[B9] Chen B. Yu L. Wang J. Li C. Zhao K. Zhang H. (2016). Involvement of prokineticin 2 and prokineticin receptor 1 in lipopolysaccharide-induced testitis in rats. Inflammation 39 (2), 534–542. 10.1007/s10753-015-0277-z 26490969

[B10] Chen Y. Wang J. Zhang Q. Xiang Z. Li D. Han X. (2017). Microcystin-leucine arginine exhibits immunomodulatory roles in testicular cells resulting in orchitis. Environ. Pollut. 229, 964–975. 10.1016/j.envpol.2017.07.081 28765008

[B11] Cook I. H. Evans J. Maldonado-Perez D. Critchley H. O. Sales K. J. Jabbour H. N. (2010). Prokineticin-1 (PROK1) modulates interleukin (IL)-11 expression via prokineticin receptor 1 (PROKR1) and the calcineurin/NFAT signalling pathway. Mol. Hum. Reprod. 16 (3), 158–169. 10.1093/molehr/gap084 19801577 PMC2816169

[B12] Dou D. Liang J. Zhai X. Li G. Wang H. Han L. (2021). Oxytocin signalling in dendritic cells regulates immune tolerance in the intestine and alleviates DSS-induced colitis. Clin. Sci. (Lond). 135 (4), 597–611. 10.1042/CS20201438 33564880

[B13] Ebokaiwe A. P. Ijomone O. M. Osawe S. O. Chukwu C. J. Ejike C. Zhang G. (2018). Alteration in sperm characteristics, endocrine balance and redox status in rats rendered diabetic by streptozotocin treatment: attenuating role of Loranthus micranthus. Redox Rep. 23 (1), 194–205. 10.1080/13510002.2018.1540675 30376784 PMC6748702

[B14] Ekaluo U. Ikpeme E. Udokpoh A. (2009). Sperm head abnormality and mutagenic effects of aspirin, paracetamol and caffeine containing analgesics in rats. Internet J. Toxicol. 7 (1), 1–9.

[B15] Eldesoqui M. Ahmed M. E. Abdel-Kareem M. A. Badawy M. M. Dawood A. F. Mohamed A. S. (2023). Curcumin mitigates malathion-induced renal injury: suppression of apoptosis and modulation of NF-κβ/TNF-α and Nrf2, and HO-1 signaling. Metabolites 13 (11), 1117. 10.3390/metabo13111117 37999213 PMC10673029

[B16] Eldesoqui M. Eldken Z. H. Mostafa S. A. Al-Serwi R. H. El-Sherbiny M. Elsherbiny N. (2022). Exercise augments the effect of SGLT2 inhibitor dapagliflozin on experimentally induced diabetic cardiomyopathy, possible underlying mechanisms. Metabolites 12 (7), 635. 10.3390/metabo12070635 35888760 PMC9315877

[B17] El-Shalofy A. S. Hedia M. G. (2021). Exogenous oxytocin administration improves the testicular blood flow in rams. Andrologia 53 (10), e14193. 10.1111/and.14193 34309888

[B18] Fijak M. Schneider E. Klug J. Bhushan S. Hackstein H. Schuler G. (2011). Testosterone replacement effectively inhibits the development of experimental autoimmune orchitis in rats: evidence for a direct role of testosterone on regulatory T cell expansion. J. Immunol. 186 (9), 5162–5172. 10.4049/jimmunol.1001958 21441459

[B19] Fırat F. Erdemir F. Kölükçü E. Gevrek F. Benli İ. Ünsal V. (2018). Oxytocin for preventing injury due to testicular torsion/detorsion in rats. Turkish J. Trauma Emerg. Surgery/Ulusal Travma ve Acil Cerrahi Dergisi. 24 (2), 89–96. 10.5505/tjtes.2017.25730 29569694

[B20] Franchi S. Giannini E. Lattuada D. Lattanzi R. Tian H. Melchiorri P. (2008). The prokineticin receptor agonist Bv8 decreases IL-10 and IL-4 production in mice splenocytes by activating prokineticin receptor-1. BMC Immunol. 9, 60. 10.1186/1471-2172-9-60 18957080 PMC2584092

[B21] Frayne J. Townsend D. Nicholson H. D. (1996). Effects of oxytocin on sperm transport in the pubertal rat. J. Reprod. Fertil. 107 (2), 299–306. 10.1530/jrf.0.1070299 8882297

[B22] Ghasemnezhad R. Mohammadghasemi F. Faghani M. Bahadori M. H. (2015). Oxytocin can decrease germ cells apoptotic index in testis under acute ischemia reperfusion in a rat model. Iran. J. reproductive Med. 13 (5), 283–290. PMC4515235 26221127

[B23] Guo Q. Cheng Y. Li T. Huang J. Li J. Zhang Z. (2024). The gut microbiota contributes to the development of LPS-induced orchitis by disrupting the blood-testosterone barrier in mice. Reprod. Sci. 31 (11), 3379–3390. 10.1007/s43032-024-01613-9 38858330

[B24] Han X. X. Jiang Y. P. Liu N. Wu J. Yang J. M. Li Y. X. (2019). Protective effects of Astragalin on spermatogenesis in streptozotocin-induced diabetes in male mice by improving antioxidant activity and inhibiting inflammation. Biomed. Pharmacother. 110, 561–570. 10.1016/j.biopha.2018.12.012 30537673

[B25] Hedger M. P. Meinhardt A. (2003). Cytokines and the immune-testicular axis. J. Reprod. Immunol. 58 (1), 1–26. 10.1016/s0165-0378(02)00060-8 12609522

[B26] Hong C. Y. Park J. H. Ahn R. S. Im S. Y. Choi H. S. Soh J. (2004). Molecular mechanism of suppression of testicular steroidogenesis by proinflammatory cytokine tumor necrosis factor alpha. Mol. Cell Biol. 24 (7), 2593–2604. 10.1128/mcb.24.7.2593-2604.2004 15024051 PMC371106

[B27] Houshmand F. Faghihi M. Zahediasl S. (2009). Biphasic protective effect of oxytocin on cardiac ischemia/reperfusion injury in anaesthetized rats. Peptides 30 (12), 2301–2308. 10.1016/j.peptides.2009.09.010 19761809

[B28] Huang C. Zhang W. Sun A. Zhang X. Guo J. Ji R. (2020). Methane ameliorates lipopolysaccharide-induced acute orchitis by anti-inflammatory, antioxidative, and antiapoptotic effects via regulation of the PK2/PKR1 pathway. Oxid. Med. Cell Longev. 2020, 7075836. 10.1155/2020/7075836 32922653 PMC7453259

[B29] Inoue T. Aoyama-Ishikawa M. Uemura M. Yamashita H. Koga Y. Terashima M. (2020). Interleukin-18 levels and mouse Leydig cell apoptosis during lipopolysaccharide-induced acute inflammatory conditions. J. Reprod. Immunol. 141, 103167. 10.1016/j.jri.2020.103167 32629316

[B30] Johnsen S. G. (1970). Testicular biopsy score count–a method for registration of spermatogenesis in human testes: normal values and results in 335 hypogonadal males. Hormone Res. Paediatr. 1 (1), 2–25. 10.1159/000178170 5527187

[B31] Jurek B. Neumann I. D. (2018). The oxytocin receptor: from intracellular signaling to behavior. Physiol. Rev. 98 (3), 1805–1908. 10.1152/physrev.00031.2017 29897293

[B32] Kawai T. Akira S. (2010). The role of pattern-recognition receptors in innate immunity: update on Toll-like receptors. Nat. Immunol. 11 (5), 373–384. 10.1038/ni.1863 20404851

[B33] Klein B. Haggeney T. Fietz D. Indumathy S. Loveland K. L. Hedger M. (2016). Specific immune cell and cytokine characteristics of human testicular germ cell neoplasia. Hum. Reprod. 31 (10), 2192–2202. 10.1093/humrep/dew211 27609978

[B34] LeCouter J. Ferrara N. (2003). EG-VEGF and Bv8. a novel family of tissue-selective mediators of angiogenesis, endothelial phenotype, and function. Trends Cardiovasc Med. 13 (7), 276–282. 10.1016/s1050-1738(03)00110-5 14522467

[B35] Leisegang K. Henkel R. Agarwal A. (2019). Obesity and metabolic syndrome associated with systemic inflammation and the impact on the male reproductive system. Am. J. Reprod. Immunol. 82 (5), e13178. 10.1111/aji.13178 31373727

[B36] Leslie S. Soon-Sutton T. Khan M. A. (2024). Male infertility. StatPearls, 194–200. 10.1201/9781003579038-20 32965929

[B37] Li L. Ma P. Liu Y. Huang C. O W. S. Tang F. (2013). Intermedin attenuates LPS-induced inflammation in the rat testis. PLoS One 8 (6), e65278. 10.1371/journal.pone.0065278 23750251 PMC3672160

[B38] Li Y. Zhan M. Li J. Zhang W. Shang X. (2023). Lycopene alleviates lipopolysaccharide-induced testicular injury in rats by activating the PPAR signaling pathway to integrate lipid metabolism and the inflammatory response. Transl. Androl. Urol. 12 (2), 271–285. 10.21037/tau-22-864 36915878 PMC10006007

[B39] Martucci C. Franchi S. Giannini E. Tian H. Melchiorri P. Negri L. (2006). Bv8, the amphibian homologue of the mammalian prokineticins, induces a proinflammatory phenotype of mouse macrophages. Br. J. Pharmacol. 147 (2), 225–234. 10.1038/sj.bjp.0706467 16299550 PMC1615858

[B40] Matsuura T. Kawasaki M. Sakai A. Ueta Y. (2015). Posterior pituitary hormones and pain. Interdiscip. Inf. Sci. 21 (3), 207–212. 10.4036/iis.2015.b.05

[B41] Mehdi S. F. Pusapati S. Khenhrani R. R. Farooqi M. S. Sarwar S. Alnasarat A. (2022). Oxytocin and related peptide hormones: candidate anti-inflammatory therapy in early stages of sepsis. Front. Immunol. 13, 864007. 10.3389/fimmu.2022.864007 35572539 PMC9102389

[B42] Metukuri M. R. Reddy C. M. Reddy P. R. Reddanna P. (2010). Bacterial LPS-mediated acute inflammation-induced spermatogenic failure in rats: role of stress response proteins and mitochondrial dysfunction. Inflammation 33 (4), 235–243. 10.1007/s10753-009-9177-4 20087639

[B43] Mulero M. C. Huxford T. Ghosh G. (2019). NF-κB, IκB, and IKK: integral components of immune system signaling. Struct. Immunol. 1172, 207–226. 10.1007/978-981-13-9367-9_10 31628658

[B44] Nicholson H. D. Greenfield H. M. Frayne J. (1994). The effect of germ cell complement on the presence of oxytocin in the interstitial and seminiferous tubule fluid of the rat testis. J. Endocrinol. 143 (3), 471–478. 10.1677/joe.0.1430471 7836892

[B45] O’Bryan M. K. Schlatt S. Phillips D. J. de Kretser D. M. Hedger M. P. (2000). Bacterial lipopolysaccharide-induced inflammation compromises testicular function at multiple levels *in vivo* . Endocrinology 141 (1), 238–246. 10.1210/endo.141.1.7240 10614644

[B46] Petrella C. Spaziani M. D'Orazi V. Tarani L. Terracina S. Tarani F. (2022). Prokineticin 2/PROK2 and male infertility. Biomedicines 10 (10), 2389. 10.3390/biomedicines10102389 36289651 PMC9598863

[B47] Reddy M. M. Mahipal S. V. Subhashini J. Reddy M. C. Roy K. R. Reddy G. V. (2006). Bacterial lipopolysaccharide-induced oxidative stress in the impairment of steroidogenesis and spermatogenesis in rats. Reprod. Toxicol. 22 (3), 493–500. 10.1016/j.reprotox.2006.03.003 16644180

[B48] Rival C. Theas M. S. Guazzone V. A. Lustig L. (2006). Interleukin-6 and IL-6 receptor cell expression in testis of rats with autoimmune orchitis. J. Reprod. Immunol. 70 (1-2), 43–58. 10.1016/j.jri.2005.10.006 16458979

[B49] Sayed M. M. Abd El-Rady N. M. Gomaa W. M. S. Hosny A. Gomaa A. M. S. (2023). Antioxidant, antiapoptotic, and antifibrotic abilities of L-Arginine ameliorate the testicular dysfunction in diabetic rats. Tissue Cell 82, 102036. 10.1016/j.tice.2023.102036 36841127

[B50] Sever I. Ozkul B. Erisik Tanriover D. Ozkul O. Elgormus C. Gur S. (2021). Protective effect of oxytocin through its anti-inflammatory and antioxidant role in a model of sepsis-induced acute lung injury: demonstrated by CT and histological findings. Exp. lung Res. 47 (9), 426–435. 10.1080/01902148.2021.1992808 34665057

[B51] Shen N. Wang Z. Wang C. Zhang J. Liu C. (2020). Methane alleviates inflammation and apoptosis of dextran sulfate sodium-induced inflammatory bowel diseases by inhibiting toll-like receptor 4 (TLR4)/Myeloid differentiation factor 88 (MyD88)/Nuclear translocation of nuclear factor-κb (NF-κB) and endoplasmic reticulum stress pathways in mice. Med. Sci. Monit. Int. Med. J. Exp. Clin. Res. 26, e922248. 10.12659/MSM.922248 PMC7297035 32500859

[B52] Shen P. Ji S. Li X. Yang Q. Xu B. Wong C. K. C. (2022). LPS-induced systemic inflammation caused mPOA-FSH/LH disturbance and impaired testicular function. Front. Endocrinol. (Lausanne) 13, 886085. 10.3389/fendo.2022.886085 35813649 PMC9259990

[B53] Syriou V. Papanikolaou D. Kozyraki A. Goulis D. G. (2018). Cytokines and male infertility. Eur. Cytokine Netw. 29 (3), 73–82. 10.1684/ecn.2018.0412 30547889

[B54] Szeto A. Sun-Suslow N. Mendez A. J. Hernandez R. I. Wagner K. V. McCabe P. M. (2017). Regulation of the macrophage oxytocin receptor in response to inflammation. Am. J. Physiol. Endocrinol. Metab. 312 (3), E183-E189–E9. 10.1152/ajpendo.00346.2016 28049625 PMC5374296

[B55] Takeda K. Akira S. (2015). Toll-like receptors. Curr. Protoc. Immunol. 109 (1), 1421–20. 10.1002/0471142735.im1412s109 25845562

[B56] Tang Y. Shi Y. Gao Y. Xu X. Han T. Li J. (2019). Oxytocin system alleviates intestinal inflammation by regulating macrophages polarization in experimental colitis. Clin. Sci. (Lond). 133 (18), 1977–1992. 10.1042/CS20190756 31519790

[B57] Thackare H. Nicholson H. D. Whittington K. (2006). Oxytocin—its role in male reproduction and new potential therapeutic uses. Hum. Reprod. Update 12 (4), 437–448. 10.1093/humupd/dmk002 16436468

[B58] Theas M. S. Jacobo P. V. Pérez C. V. Guazzone V. A. Lustig L. (2018). “Inflammation and spermatogenesis,” in Spermatogenesis (CRC Press), 63–72.

[B59] Theas M. S. Rival C. Jarazo-Dietrich S. Jacobo P. Guazzone V. A. Lustig L. (2008). Tumour necrosis factor-alpha released by testicular macrophages induces apoptosis of germ cells in autoimmune orchitis. Hum. Reprod. 23 (8), 1865–1872. 10.1093/humrep/den240 18579514

[B60] Tsubaki M. Takeda T. Kino T. Itoh T. Imano M. Tanabe G. (2015). Mangiferin suppresses CIA by suppressing the expression of TNF-α, IL-6, IL-1β, and RANKL through inhibiting the activation of NF-κB and ERK1/2. Am. J. Transl. Res. 7 (8), 1371–1381. 26396668 PMC4568793

[B61] Uvnäs-Moberg K. Ekström-Bergström A. Berg M. Buckley S. Pajalic Z. Hadjigeorgiou E. (2019). Maternal plasma levels of oxytocin during physiological childbirth–a systematic review with implications for uterine contractions and central actions of oxytocin. BMC pregnancy 19, 285–317. 10.1186/s12884-019-2365-9 PMC6688382 31399062

[B62] Wang F. Liu W. Jiang Q. Gong M. Chen R. Wu H. (2019a). Lipopolysaccharide-induced testicular dysfunction and epididymitis in mice: a critical role of tumor necrosis factor alpha†. Biol. Reprod. 100 (3), 849–861. 10.1093/biolre/ioy235 30398566

[B63] Wang G. Xu B. Shi F. Du M. Li Y. Yu T. (2019b). Protective effect of methane-rich saline on acetic acid-induced ulcerative colitis via blocking the TLR4/NF-κB/MAPK pathway and promoting IL-10/JAK1/STAT3-mediated anti-inflammatory response. Oxidative Med. Cell. Longev. 2019, 7850324. 10.1155/2019/7850324 PMC6512011 31182999

[B64] Yuan L. Liu S. Bai X. Gao Y. Liu G. Wang X. (2016). Oxytocin inhibits lipopolysaccharide-induced inflammation in microglial cells and attenuates microglial activation in lipopolysaccharide-treated mice. J. Neuroinflammation 13 (1), 77. 10.1186/s12974-016-0541-7 27075756 PMC4831099

[B65] Zhu C. L. Wang L. Zhao X. Q. Yang R. Zhang B. Y. Zhao Y. Y. (2022). Antimicrobial peptide MPX attenuates LPS-induced inflammatory response and blood-testis barrier dysfunction in Sertoli cells. Theriogenology 189, 301–312. 10.1016/j.theriogenology.2022.07.001 35842953

